# Prolyl hydroxylase 2 dependent and Von-Hippel-Lindau independent degradation of Hypoxia-inducible factor 1 and 2 alpha by selenium in clear cell renal cell carcinoma leads to tumor growth inhibition

**DOI:** 10.1186/1471-2407-12-293

**Published:** 2012-07-17

**Authors:** Sreenivasulu Chintala, Tanbir Najrana, Karoly Toth, Shousong Cao, Farukh A Durrani, Roberto Pili, Youcef M Rustum

**Affiliations:** 1 Department of Cancer Biology, Roswell Park Cancer Institute, Buffalo, NY, 14263, USA; 2 Department of Medicine, Roswell Park Cancer Institute, Buffalo, New York, 14263, USA; 3 Department of Pharmacology & Therapeutics, Roswell Park Cancer Institute, Buffalo, NY, 14263, USA

**Keywords:** Prolyl hydroxylases, Hypoxia-inducible factor, Clear cell renal cell carcinoma, Selenium

## Abstract

**Background:**

Clear cell renal cell carcinoma (ccRCC) accounts for more than 80% of the cases of renal cell carcinoma. In ccRCC deactivation of Von-Hippel-Lindau (VHL) gene contributes to the constitutive expression of hypoxia inducible factors 1 and 2 alpha (HIF-α), transcriptional regulators of several genes involved in tumor angiogenesis, glycolysis and drug resistance. We have demonstrated inhibition of HIF-1α by Se-Methylselenocysteine (MSC) via stabilization of prolyl hydroxylases 2 and 3 (PHDs) and a significant therapeutic synergy when combined with chemotherapy. This study was initiated to investigate the expression of PHDs, HIF-α, and VEGF-A in selected solid cancers, the mechanism of HIF-α inhibition by MSC, and to document antitumor activity of MSC against human ccRCC xenografts.

**Methods:**

Tissue microarrays of primary human cancer specimens (ccRCC, head & neck and colon) were utilized to determine the incidence of PHD2/3, HIF-α, and VEGF-A by immunohistochemical methods. To investigate the mechanism(s) of HIF-α inhibition by MSC, VHL mutated ccRCC cells RC2 (HIF-1α positive), 786–0 (HIF-2α positive) and VHL wild type head & neck cancer cells FaDu (HIF-1α) were utilized. PHD2 and VHL gene specific siRNA knockdown and inhibitors of PHD2 and proteasome were used to determine their role in the degradation of HIF-1α by MSC.

**Results:**

We have demonstrated that ccRCC cells express low incidence of PHD2 (32%), undetectable PHD3, high incidence of HIF-α (92%), and low incidence of VEGF-A compared to head & neck and colon cancers. This laboratory was the first to identify MSC as a highly effective inhibitor of constitutively expressed HIF-α in ccRCC tumors. MSC did not inhibit HIF-1α protein synthesis, but facilitated its degradation. The use of gene knockdown and specific inhibitors confirmed that the inhibition of HIF-1α was PHD2 and proteasome dependent and VHL independent. The effects of MSC treatment on HIF-α were associated with significant antitumor activity against ccRCC xenograft.

**Conclusions:**

Our results show the role of PHD2/3 in stable expression of HIF-α in human ccRCC. Furthermore, HIF-1α degradation by MSC is achieved through PHD2 dependent and VHL independent pathway which is unique for HIF-α regulation. These data provide the basis for combining MSC with currently used agents for ccRCC.

## Background

Kidney cancer is associated with several gene mutations including Von-Hippel-Lindau (VHL), fumarate hydratase (FH) and succinate dehydrogenase (SDH) [1] and accounts for 3% of all cancers with 2% of the total cancer deaths in the U.S [2]. Among all kidney cancers, clear cell renal cell carcinoma (ccRCC) is the most common with a molecularly distinct phenotype [3] and contain up to 91% nonfunctional VHL mutations [1] which may leads to the constitutive expression of hypoxia inducible factors 1 and 2 alpha (HIF-α). HIF-α are transcription factors that regulate several genes (>100) involved in angiogenesis, cell proliferation, apoptosis, glycolysis, iron metabolism, drug resistance and other cellular processes to adapt to low oxygen hypoxic conditions in various solid tumors [4]. HIF-α are susceptible to oxygen dependent degradation under regular oxygenated conditions through the hydroxylation of proline molecules by prolyl hydroxylases (PHDs). There are four isoforms of PHDs (PHD1- 4) whose functions are different in various cellular systems [5,6]. Under regular oxygen conditions, HIF-1α is hydroxylated at proline 402 and proline 564 molecules by prolyl hydroxylases 2 and 3 (PHD2/3) which is then recognized by VHL and further degraded by proteasome [7,8]. In general, PHD2 and PHD3 are expressed normally under normoxic conditions and their hydroxylation activity is inhibited under hypoxic conditions, resulting in the stable expression of HIF-α proteins [9]. The preferential hydroxylation of HIF-1α by PHD2 and HIF-2α by PHD3 has been reported [7]. Recent report, however, indicates that PHD2 and 3 hydroxylate HIF-1α and HIF-2α with similar efficiency [10]. The differences in cellular localization of PHD 2 and 3 have been reported, showing that PHD2 expression mostly in cytoplasm and PHD3 in cytoplasm and nucleus [11]. Recently, PHD3 mRNA deregulation has been reported in several cancers including renal, prostate, breast and melanoma [12]. PHD3 is not only involved in the hydroxylation of HIF-α, but also has a role in apoptosis [13,14].

Stable expression of HIF-α is regulated by synthesis and degradation pathways. It is likely that a lower level of HIF-α degradation pathway exists in ccRCC due to mutations in VHL. Several modulators of PHD activity have been implicated in HIF-stability. These include enhanced levels of reactive oxygen species (ROS) [15], nitric oxide [16,17], 2-oxoglutarate, Fe (II), ascorbate, fumarate and succinate [15]. Recent report by Finely et al., showed that decreased levels of SIRT 3, mitochondrial NAD-dependent deacetylase, resulted in increased cellular accumulation of ROS that contributes for the stability of HIF-α [18].

To date, significant advancement in treatment of ccRCC with VEGF targeted inhibitors, has provided a proof of principle that targeting HIF-α, an upstream regulator of VEGF, may result in significant therapeutic benefits [1,19,20]. As an alternative to the development of HIF-α targeting agents, the approach to develop agents that promote the degradation of HIF-α through activation of PHDs may be more productive in alleviating HIF-α signaling pathways [10]. Since the PHDs are upstream regulators of HIF-α and their role in the degradation of HIF-α in ccRCC expressing mutant VHL has not been well investigated.

Selenium is an essential element extensively utilized in prevention clinical trials, but has thus far produced conflicting results [21] . It is likely that effectiveness of selenium is influenced by the selenium dose and types. While selenomethionine (SLM) is extensively utilized in prevention trials, Se-Methylselenocysteine (MSC) is under development and offers biological and pharmacological properties not common with SLM. Our laboratory was the first to report that optimal dose and schedule of MSC as highly effective inhibitor of ROS [22], nitric oxide [23], and HIF-1α [22,23]. This inhibition was associated with anti-angiogenic effect and tumor vasculature maturation [24] leading to moderate antitumor (30%) effect. Furthermore, remarkable therapeutic synergy was achieved in human tumor xenografts when optimal dose and schedule of MSC was used in sequential combination with anticancer drugs [22,25]. We have proved HIF-1α as a critical target molecule for MSC effects demonstrating therapeutic synergy in HIF-1α knockdown xenograft tumors with anticancer agent alone as comparable to MSC combination with anticancer agent [22]. HIF-1α inhibition by siRNA also sensitized hypoxic human tumor cells to radiotherapy [26].

The current study was designed to a) investigate the incidence of PHD2/3, HIF-α in selected human solid cancers b) test the hypothesis that degradation of HIF-α by MSC is PHD2 and proteasome dependent, VHL and PHD3 independent and c) determine if these effects will translate into therapeutic benefit without toxicity in ccRCC tumor xenografts.

## Methods

### Human primary tumors

De-identified human primary tumors of ccRCC, head & neck, and colon cancers separately arranged in Tissue microarrays (TMAs) (cores 6 mm in diameter)) and available frozen tissues of ccRCC tumors with their matched normal kidney were obtained from the Data Bank and BioRepository (DBBR) core facility, Department of Pathology, Roswell Park Cancer Institute, according to the institutional review board (IRB) approved protocols.

### Chemicals and antibodies

Se-Methylselenocysteine (MSC) was obtained from Sigma-Aldrich (St. Louis, MO). Methylselenic acid (MSA), the active metabolite of MSC, was purchased from PharmaSe Inc., (Lubbock, TX). L-[^35^ S]-Methionine purchased from PerkinElmer (Waltham MA). HIF-1α antibody was procured from R & D Systems (Minneapolis, MN) and Novus Biologicals (Littleton, CO). HIF-2α antibody was purchased from Abcam (Cambridge, MA) and Novus Biologicals (Littleton, CO). PHD 2 antibody as procured from Novus Biologicals (Littleton, CO) and PHD3 was obtained from Abcam (Cambridge, MA). Proteasome inhibitor MG132 was purchased from Calbiochem (La Jolla, CA). Protein synthesis inhibitor cycloheximide (CHX) was purchased from Sigma (St. Louis, MO). Prolyl hydroxylases inhibitor dimethyloxaloyl glycine (DMOG) was obtained from Frontier Scientific Inc., (Logan, UT).

### Immunohistochemical methods

Human primary tumors and some normal tissues (10% neutral buffered formalin fixed and paraffin embedded) arranged in TMAs (core size 0.6 mm in diameter) of ccRCC (number 88), head & neck (number 210) and colorectal (number 65) were used to evaluate expression of HIF-1α and PHD2 and 3 simultaneously in ccRCC and separately for the individual markers in head & neck and colorectal cancers by the immunohistochemical method developed previously by our laboratory [27]. Briefly, paraffin sections (5 μm thickness) were cut from TMA blocks and immunostained with automatic immunostainer for HIF-1α, PHD2 and PHD3. This is a well characterized, validated, and reliable immunohistochemical method utilizing a Catalyzed Signal Amplification (CAS) reagent that made HIF-1α nucleus confined detection possible. HIF-2α was detected with the same method using anti-HIF-2α from Novus Biologicals (Littleton, CO) at a concentration of 0.5 μg/ml. Vascular endothelial growth factor-A (VEGF-A) was immunostained according to the method described by us [22] as single staining. Well known positive, negative and isotype controls were included in all immunohistochemical studies. All immunohistochemical slides were reviewed by a board certified, experienced pathologist (K.T).

### Semiquantitative assessment of the immunostainings

HIF-1α and HIF-2α immunostainings were considered specific if they were localized in the nuclei, while for PHD2 and PHD3 cytoplasmic staining were considered as specific. Staining intensity was categorized as: not detectable in any tumor cells or the specific staining in the majority of tumor cells was weak (w), moderate (m) or strong (s). Staining intensity in the cores were compared with the known strong positive controls and categorized accordingly. Since one core (0.6 mm in diameter) of the TMA contains roughly 600 tumor cells (varies based on the size of stroma, necrosis, inflammatory cell infiltration, size of vessels etc.) the estimated 1% of the total tumor cell population (in general 5–6 tumor cells at least) was considered as positive. During the original reading of the TMA’s the staining in the cores was estimated and categorized with a letter (w, m, s) indicating the staining intensity and with a number which showed the distribution of the percentage of tumor cells stained positively like: s/100 meaning 100% of the tumor cell stained strongly for the marker. The raw data were tabulated, and based on that we have provided additional data to the result section in order to better characterize the overall staining.

### Cell culture and MSA treatment

Clear cell renal cell carcinoma cells RC2 which express HIF-1α only and 786–0 cells which express HIF-2α [28] were utilized for the studies. RC2 cells were cultured in DMEM with glucose, 10% FBS, 1.0% PSN. 786–0 cells were cultured in RPMI-1640 with 10% FBS and 1% PSN. Head & Neck cancer cells FaDu were purchased from American Type Culture Collection (Manassas, VA) and cultured in RPMI-1640 supplemented with 10% FBS. Cells were regularly tested for Mycoplasma. Since the HIF-1α was not expressing constitutively in FaDu cells, 0.5% oxygen hypoxic conditions were used with a hypoxia chamber (IN VIVO 400 Ruskinn Technology Ltd., Cincinnati, OH) to induce HIF-1α as described earlier [22]. Cells were seeded in the tissue culture plates and allowed to grow to a confluence of ~70%, then treated with MSA at a dose of 1 μM for FaDu, 10 μM for RC2 and 8–10 μM for 786–0 and incubated for 8–24 h. Untreated controls were maintained for comparison.

### Effect of MSA on cell growth

Cells (400–600) were seeded in 96 well plates, allowed to grow overnight, and treated with various concentrations (3, 5, 7 and 10 μM) of MSA for 24 h. Untreated controls were maintained without treatment. Cells were washed with medium for 3 times to remove MSA, fresh medium was added and incubated at 37 °C incubator for 96 h. Growth inhibition of cells was determined by sulforhodamine-B (SRB) assay as described previously [22]. Briefly, medium was taken out and cells were fixed with 10% trichloroacetic acid and left the plates at 4 °C for at least 2 hours. Plates were processed for washing and staining with SRB using automated plate washer. The unbound SRB stain was removed by washing and the stain bound to the cells was measured using plate reader (Bio Tek Instruments, model EL340, Winooski, VT) at 570 nm. Percent growth inhibition was determined considering growth of untreated control cells as 100%.

### Effect of MSA on HIF-1α protein synthesis and degradation

To determine the MSA effect on HIF-1α protein synthesis, FaDu cells were treated with known protein synthesis inhibitor cycloheximide (CHX) alone and in combination with MSA for 1–24 h under 0.5% hypoxic conditions because HIF-1α degradation was observed after 18-24 h of MSA exposure in these cells. To investigate the half-life of HIF-1α, RC2 cells were treated with CHX for 2, 4 and 8 h time points and evaluated HIF-1α inhibition in these cells. Further studies to evaluate the effect of MSA on HIF-1α protein synthesis, RC2 cells were treated with CHX with and without MSA for 8 h because at this earliest time point we found pronounced HIF-1α inhibition by MSA in RC2 cells. Total protein extracts were isolated and determined the HIF-1α expression by western blot. To further determine MSA effects on total protein synthesis, RC2 cells were pulsed with ^35^ S-methionine for 1 h before the 5 h incubation with 10 μM MSA or 5 μM CHX as described [29]. Total protein extracts (20 μg) were used to determine the incorporated radiolabeled methionine by SDS polyacrylamide gel electrophoresis followed by autoradiography. Gel was stained with coomassie blue stain and showed as loading control.

Total ^35^ S-methionine incorporated in the proteins was also determined by counting the radioactivity present in the protein extracts using Beckman LS 6000 Scintillation Counter. Total number of counts was calculated in one milligram of protein and compared with untreated controls. To investigate the effect of MSA on proteasome mediated degradation of HIF-1α, FaDu cells were treated with MSA and proteasome inhibitor N-[(phenylmethoxy)carbonyl]-L-leucyl-N-[(1 S)-1-formyl-3-methylbutyl]-L-leucinamide, (MG132) alone and in combination, and the HIF-1α protein level was determined by western blot analysis. The effect of MG132 on the degradation of HIF-1α in RC2 cells was determined by treating cells with MSA and MG132 alone and in combination concurrently and pretreatment of MG132 1 h before treating with MSA for 8 h. Protein extracts were prepared from the cells and used for determining HIF-1α expression by western blot.

### PHDs inhibition by dimethyloxallyl glycine (DMOG)

PHDs activity inhibitor, DMOG was used to treat cells with and without MSA to determine the HIF-1α degradation effects of MSA. FaDu which do not express HIF-1α under normoxic culture conditions were treated separately with 0.5 mM DMOG alone and in combination with MSA for 18–24 h. Cells were processed for extraction of protein and western blot was performed to measure the HIF-1α levels. Similarly, RC2 cells which express HIF-1α constitutively were treated with 0.5 mM DMOG and 10 μM MSA alone and in combination and determined the HIF-1α levels in these cells.

### SiRNA transfection

To determine the PHD2 role in the degradation of HIF-1α by MSA, RC2 cells expressing PHD2 were utilized to knockdown PHD2. To evaluate whether MSA is utilizing VHL independent pathway of degradation of HIF-1α, FaDu cells which express wild type VHL were used to knockdown VHL by siRNA. Since RC2 cells express mutated VHL we have used FaDu cells for VHL knockdown experiments. Validated Silencer sure siRNA for the egg-laying-defective nine 1 (EGLN1) gene for PHD2 protein was purchased from Ambion/Invitrogen (Carlsbad, CA). VHL SMART pool siRNA was purchased from Thermo Scientific (Lafayette, CO). Cells were allowed to grow overnight to reach ~70-80% confluence and siRNA transfection was performed using a Lipofectamine 2000 transfection reagent as per the procedure described by the manufacturer (Invitrogen, Carlsbad, CA). Briefly 200-500nM of siRNA was used with Lipofectamine 2000 and transfected into the cells and incubated at 37 °C, 5% CO_2_ for 24 h. Cells were trypsinized and seeded onto new tissue culture dishes and allowed to grow for 24–48 h. Cells were treated with and without MSA for 18–24 h and processed for the extraction of protein to determine the VHL, PHD2 and HIF-1α levels by western blot. Each experiment was repeated at least twice.

### Western blot analysis

Western blot analysis was performed to determine the effect of MSA or MSC on HIF-α, and PHDs as per the procedure described previously [22]. Briefly, after the treatments, cells were washed twice with PBS, scrapped with a cell scrapper, centrifuged and cell pellets were collected. Protein extracts were prepared from the cell pellets using the lysis buffer with protease inhibitors and brief sonication. Tumor xenografts and human primary tumor tissues were collected, and snap-frozen in liquid nitrogen. Protein extracts were prepared by homogenizing with a Polytron homogenizer in lysis buffer. Twenty to forty μg of protein was used to separate on high efficient Mini-Protean precast 4-20% gradient gel (Bio-Rad) and transfer to the PVDF membrane. Primary antibodies for HIF-1α (R and D systems 1:250/500 dilution), HIF-2α (Abcam, 1:250/500 dilution) PHD2 (Novus Biologicals 1:5000 dilution), PHD3 (Abcam 1:500 dilution), and VHL (Cell Signaling 1:500 dilution) were used and incubated for 1 h at room temperature or overnight at 4 °C. Respective HRP conjugated secondary antibodies were used and incubated for 1 h. Proteins were detected using Lumi-Light PLUS western blotting kit (Roche, Indianapolis, IN) for HIF-1α, PHD2/3 and VHL and an ECL advance kit for HIF-2α.

### Vascular endothelial growth factor (VEGF) analysis by enzyme-linked immunosorbent assay (ELISA)

RC2 and 786–0 cells were seeded in 6 well plates and allowed to grow overnight in a regular culture medium. The cell culture medium was aspirated and fresh medium was added with reduced serum (1% FBS) and treated with MSA for 24 h. Cell culture supernatants from untreated and MSA treated cells were collected, centrifuged and immediately used for measuring secreted VEGF using a Quantikine Human VEGF Immunoassay kit as per the manufacturer’s instructions (R & D Systems, Minneapolis, MN). Briefly, 50 μl of Assay Diluent was added to each well. Plate layout was marked with standard, control and experiment and 200 μl of VEGF standard, cell culture supernatants of control and experiment were added and incubated for 2 h at room temperature. Each well was aspirated and washed 3 times with wash buffer and 200 μl of VEGF conjugate was added and incubated for 2 h at room temperature. Aspiration and washing was repeated 3 times and 200 μl substrate solution was added to each well, the plate was protected from light and incubated for 20 min at room temperature. Reaction was stopped by adding 50 μl stop solution and mixing the plate gently, optical density was recorded at 450 nm using a microplate reader with correction at 540 nm. The concentration of secreted VEGF was calculated using the standard curve created by plotting the mean absorbance on y-axis against the concentration on the x-axis.

### RT-PCR analysis

The expression of HIF-1α and PHD2/3 were determined by quantitative real time PCR (pRT-PCR) analysis as per the methods described earlier [30] Total RNA was isolated from ccRCC cells and primary tumor tissues with matched adjacent normal kidney using the TRIzol method (Invitrogen, Carlsbad, CA). Complementary DNA (cDNA) was synthesized from total RNA using a Superscript First-strand synthesis kit according to the manufacturer’s instructions (Invitrogen, Carlsbad, CA). For quantitative analysis of expression of HIF-1α and PHD2/3, qRT-PCR was performed with SYBR green quantitative PCR technique using the Applied Biosystems Real-Time Cycler HT-7900. Expression levels were normalized to β-actin mRNA levels by calculating delta cycle thresholds (ΔCt) (ΔCt = Ct of the gene (HIF-1α, PHD2/3) – Ct of β-actin). Relative mRNA expression for each gene was normalized to control normal kidney tissues by using 2delta-delta CT method as described by manufacturer (Applied Biosystems). For determining the expression of genes in ccRCC cells the average delta CT values normalized to endogenous β-actin control were used to show the expression levels of genes in each cell line. Experiments were performed with replicate samples.

### Nude mice

Female athymic NUDE-Foxn1 (nu/nu) mice, 8–12 weeks old (weight 20-25 g) were purchased from Harlan Sprague- Dawley Inc., (Indianapolis, IN). Mice were kept five per cage with water and food ad libitum according to the protocols approved by the Institute Animal Care and Use Committee (IACUC) at Roswell Park Cancer Institute.

### Tumor measurement and antitumor activity

Vernier Caliper was used to measure the two axis (mm) of tumor (L, longest axis; W, shortest axis). The weight of the tumor was estimated using the formula: tumor weight = ½ (L x W^2^). Tumor measurements were taken daily for the first 8 days and at least 3 times each week for the following 2 weeks. Antitumor activity of selenium was determined by assessing the tumor size. Animals were sacrificed when the tumor weight reached ~2 grams according to the Institute’s approved animal protocols.

### Statistical analysis

Statistical analysis was performed using GraphPad Prism Software Inc. (La Jolla, CA). Standard Student’s *t*-test was used to determine the significance between untreated control and selenium treatments and p < 0.05 was considered as significant. To determine whether the incidence of PHD2/3, HIF-α and VEGF in ccRCC is significantly different from head & neck and colon cancer, the data was analyzed by Dr. Austin Miller (Senior Biostatistician, Department of Biostatistics, at Roswell Park Cancer Institute). Estimates and 95 percent confidence limits for the proportion of tissue sample with positive expression were calculated using Wilson Point Estimation methods [31]. Statistical significance for the difference in expression was assessed using Fishers Exact test. This test provides a measure of evidence supporting the null hypothesis of no difference in expression in a given marker between the disease groups (compared to ccRCC).

## Results

### Low incidence of PHD2 and VEGF-A, undetectable PHD3, and high incidence of HIF-α, in human ccRCC tumors compared to head & neck and colon cancers

**Figure 1:**
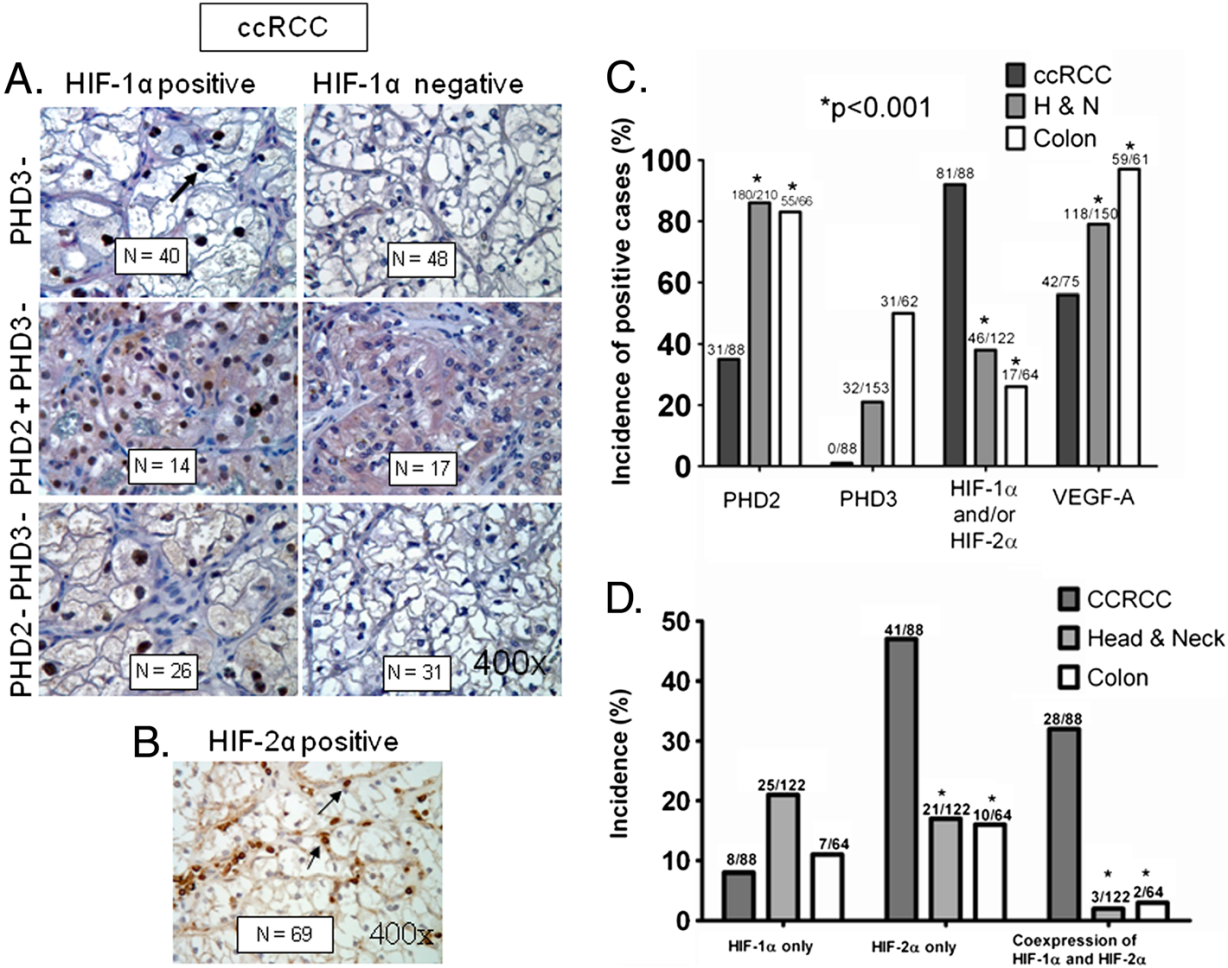
**Incidence of PHD2/3, HIF-α, and VEGF-A in ccRCC, head & neck and colon primary cancers.** (**A**) Double immunohistochemical detection of HIF-1α and PHD2/3 in TMAs of ccRCC. Representative photomicrographs (all magnification x400) of HIF-1α positive and HIF-1α negative tumors (upper panel) showing nuclear staining of HIF-1α. Arrow indicates brown nuclear staining. Representative photomicrographs of PHD2 positive and PHD3 negative tumors (pink cytoplasmic staining, middle panel) and PHD2/3 negative tumors showing no cytoplasmic pink staining (lower panel). Numbers shown in the boxes are the positive/negative tumors. (**B**) Representative photomicrograph (x400) of HIF-2α positive tumors in TMA of ccRCC. Arrows indicate brown nuclear staining. (**C**) Percent incidence of PHD2, PHD3, HIF-α (HIF-1α and/or HIF-2α), and VEGF-A in ccRCC, head & neck, and colon primary tumor biopsies arranged in TMA. Numbers at the top of column indicate the number of positive cases among all evaluable cases. (**D**) Incidence of exclusively HIF-1α, HIF-2α and both HIF-1α and HIF-2α positive cases from the primary cancers in TMA. Some of the HIF-1α positive tumors turned out to be positive for HIF-2α and vice versa for HIF-2α positive tumors, which were excluded to count only HIF-1α, HIF-2α, and both HIF-1α and HIF-2α positive cases. The co-expression of HIF-1α and HIF-2α together was also significantly higher in ccRCC as compared to head & neck and colon cancers. Fisher Exact test revealed the statistically significant difference of incidence in ccRCC when compared to head & neck and colon cancers.*P < 0.001.

To determine the potential clinical relevance of the expression of PHD 2/3, HIF-α and VEGF-A proteins and their modulation by therapeutic doses of MSC, we have evaluated their incidence, intensity and cellular distribution in ccRCC (n = 88), head & neck (n = 210), and colorectal (n = 65) human primary cancer specimens. Cancer specimens arranged in TMA were utilized to evaluate the markers simultaneously in the same cells by double immunohistochemical methods for HIF-α and PHD2 or PHD3 as described earlier [27]. As shown in Figure 1A and 1B, specific nuclear staining of HIF-1α and HIF-2α (arrows, brown) and cytoplasmic PHD2 (middle panel, pink) were found in ccRCC samples. PHD3 protein was undetectable (lower panel) in all 88 tumors. The percent incidence of these markers presented in Figure 1C shows 35% PHD2, no detectable PHD3, 92% of HIF-α (HIF-1α and/or HIF-2α), and 56% of VEGF-A in 88 cases of ccRCC. Some of the HIF-1α positive tumors were also positive for HIF-2α and vice versa for HIF-2α expressing tumor. Tumors positive for HIF-2α were excluded to determine exclusively HIF-1α incidence and vice versa for HIF-2α incidence. The data presented in Figure 1D show that the incidence of HIF-1α only (9%) was significantly low compared to HIF-2α only (47%) and co-expression of HIF-1α and HIF-2α (32%) in ccRCC. In most cases, the nuclear staining intensity was strong for both HIF-1α and HIF-2α. Cytoplasmic staining was weak for PHD2 and VEGF-A. The data in Figure 1A-D demonstrated that the overall incidence and protein expression of HIF-2α were dominant compared to HIF-1α in ccRCC tumors. 

HIF-1α staining intensity was strong in all samples of ccRCC, and the average distribution was 66% (in 13 cases even all tumor cells nuclei expressed HIF-1α) but the incidence (presence) of HIF-1α alone was 9%. This 9% was significantly lower than HIF-2α alone (47%). In head & neck and colorectal cancers HIF-1α staining was less intense (weak to moderate intensity) and involved in smaller areas. HIF-2α distribution in ccRCC, head & neck, and colorectal cancer are 15%, 5%, and 11% respectively, meaning relatively few tumor cells express HIF-2α in positive cases. Incidence of HIF-2α only in ccRCC is relatively high (46%) but in these positive samples, generally few tumor cell nuclei (15%) express HIF-2α. The average distribution of PHD2 in ccRCC was 64% with weak intensity, while in head & neck and colorectal cancers PHD2 was expressed very uniformly, almost in all tumor cells (98% and 95% average distribution) with variable staining intensity (from weak to strong). PHD3 was not detectable in any sample of ccRCC. In contrast to ccRCC, in head & neck and colorectal cancers, the majority of tumor cells (84% and 78% respectively) express PHD3 from weak to moderate intensity.

Head & neck and colon cancers have significantly high incidence of PHD2 (86% in head & neck, and 83% in colon) and PHD3 (21% in head & neck, and 50% in colon), and low incidence of HIF-α (38% in head & neck, and 27% in colon) (Figure 1C) compared to ccRCC. Despite the low incidence of HIF-α, the incidence of VEGF-A was found to be 79% and 97% in head & neck and colon tumors, respectively (Figure 1C). Determination of HIF-1α only, HIF-2α only, and co-expression of HIF-1α & HIF-2α revealed that the incidence of HIF-1α only was high (20%) in head & neck cancer compared to colon (11%) and ccRCC (9%), whereas HIF-2α only incidence was low in head & neck (17%) and colon (16%) cancers compared to ccRCC (47%). The co-expression incidence of HIF-1α and HIF-2α was very low in head & neck (2%) and colon (3%) cancers compared to ccRCC (32%) (Figure 1D). Collectively, these data suggest that an inverse relationship trend between HIF-α incidence and PHDs expression in ccRCC, head & neck and colon cancers. Furthermore, the findings also revealed high incidence of HIF-2α and co-expression of HIF-1α and HIF-2α in ccRCC compared to head & neck and colon cancers. The data presented in Table 1 is a tabulation of the incidence ratio of HIF-1α, HIF-2α to PHD2 and PHD3. The data indicate that the ratios of HIF-α to PHD2 in ccRCC were approximately 5–17 fold higher than that of head & neck and colon tumors.

**Figure 2:**
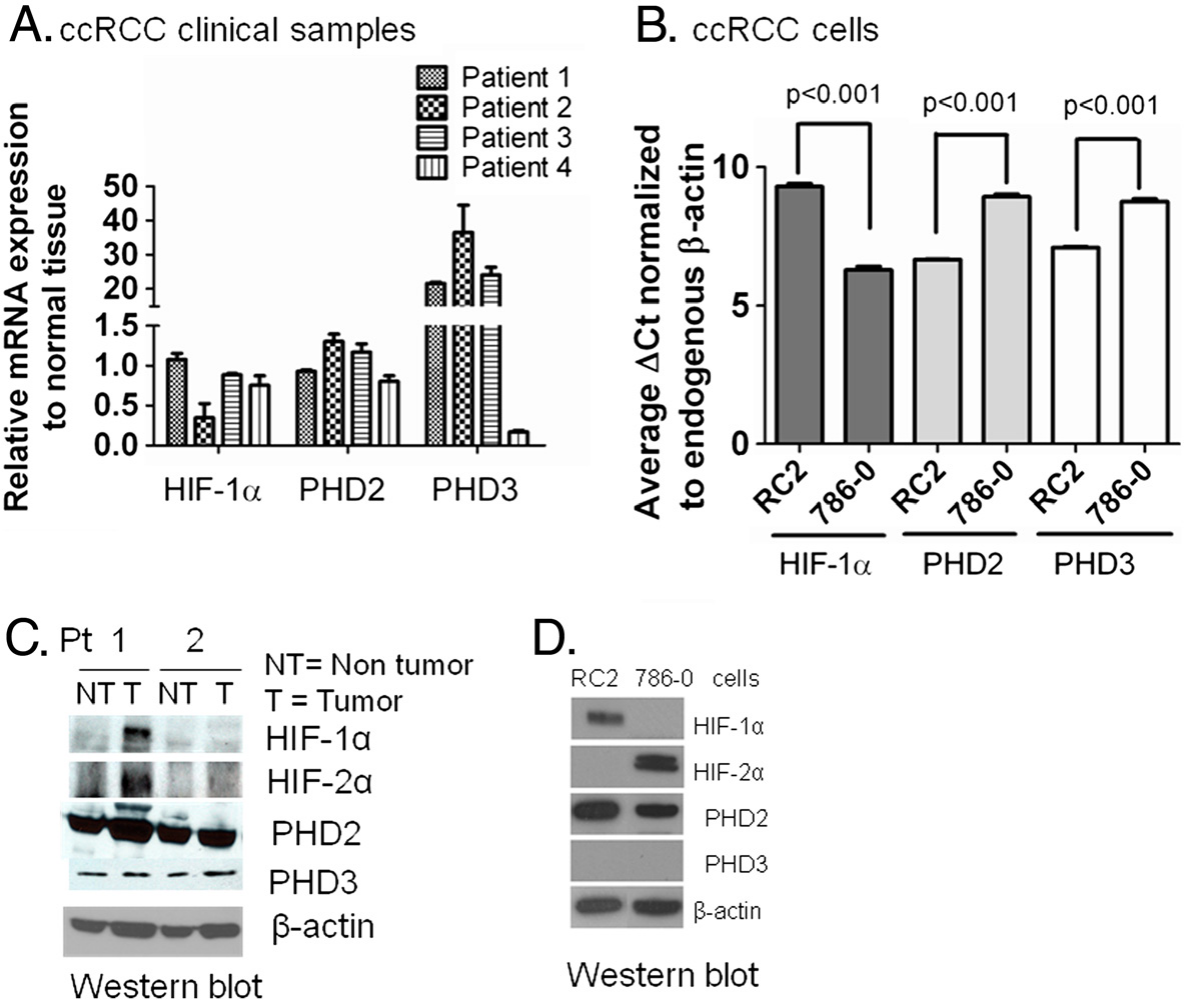
**Expression of HIF-α, PHD2, and PHD3 mRNA and protein in ccRCC clinical specimens and cell lines.** (**A**) Quantitative analysis of HIF-1α, PHD2, and PHD3 mRNA by real-time RT-PCR (qRT-PCR) in ccRCC primary tumors. Expressions were normalized to the matched normal kidney tissue by calculating ^2delta-deltaCT^ values relative to normal kidney reference. Expression of mRNA in individual tumors was shown. **B**. Expression analysis of HIF-1α, PHD2, and PHD3 in ccRCC cells RC2 and 786–0. Expression was normalized to endogenous β-actin by calculating delta cycle threshold (ΔCt). ΔCt = Ct value of specific gene (HIF-1α, PHD2 and PHD3) - Ct value of β-actin. The lower the ΔCt value the higher the expression of the gene. Experiment was repeated twice with triplicates and p < 0.05 was considered as significant P < 0.001. (**C**) Detection of HIF-1α, HIF-2α and PHD2/3 in ccRCC primary tumors and their matched normal kidney tissues by western blot analysis. 80 micrograms of protein extract was electrophoresed through Mini-Protean precast 4-20% gradient gel. Expression of β-actin was used as a loading control. (**D**) PHD3 protein was undetectable in ccRCC cells. Detection of HIF-α and PHD2/3 by western blot analysis in ccRCC cells RC2 and 786–0 cells. β-actin expression was used as a loading control.

**Table 1:** Ratio of HIF-α to PHDs incidence in solid tumor specimens

**Incidence ratio**^@^				
	**HIF-1α/PHD2**	**HIF-1α/PHD3**	**HIF-2α/PHD2**	**HIF-2α/PHD3**
ccRCC	1.29^*^	-	2.22^*^	-
H & N	0.22	1.25	0.13	0.72
Colorectal	0.15	0.26	0.18	0.32

^@^Incidence ratio was calculated by dividing incidence of HIF-α by the incidence of PHD2 or PHD3. ^*^P < 0.01.

### CCRCC cell lines express similar HIF-α and PHDs profiles as in clinical samples

Since PHD3 protein was undetectable in 88 ccRCC tumors (Figure 1A and C), we have investigated the expression of PHD 2/3 mRNA and protein in selected clinical samples and ccRCC cell lines. The data in Figure 2A show the expression of PHD2, 3 and HIF-1α mRNA in primary tumors. Quantitative real time RT-PCR (qRT-PCR) analysis revealed the normal expression of HIF-1α, PHD2 and significantly high expression of PHD3 mRNA (3 out of 4) in primary tumors compared to their matched normal kidney (Figure 2A). There was variability in the expression of these markers among the tumors. In accordance with the clinical samples, the ccRCC cell lines RC2 and 786–0 expresses mRNA of HIF-1α and PHD2/3 (Figure 2B). Like in primary tumor tissues there was a difference in the expression levels of these genes in the two cells lines. However, PHD3 protein was undetectable in 88 tumor tissues by immunohistochemistry (Figure 1C) and in two cell lines (Figure 2D). A very weak expression of PHD3 was found by western blot analysis in tumor tissues (Figure 2C), likely derived from stromal cells since the whole tumor extract was used to do western blot analysis. The ccRCC cells RC2 and 786–0 used to determine mechanism of HIF-1α regulation by PHDs have similar molecular profile like clinical samples expressing PHD2 protein and deficient in PHD3 protein but not mRNA (Figure 2B and D).

**Figure 3:**
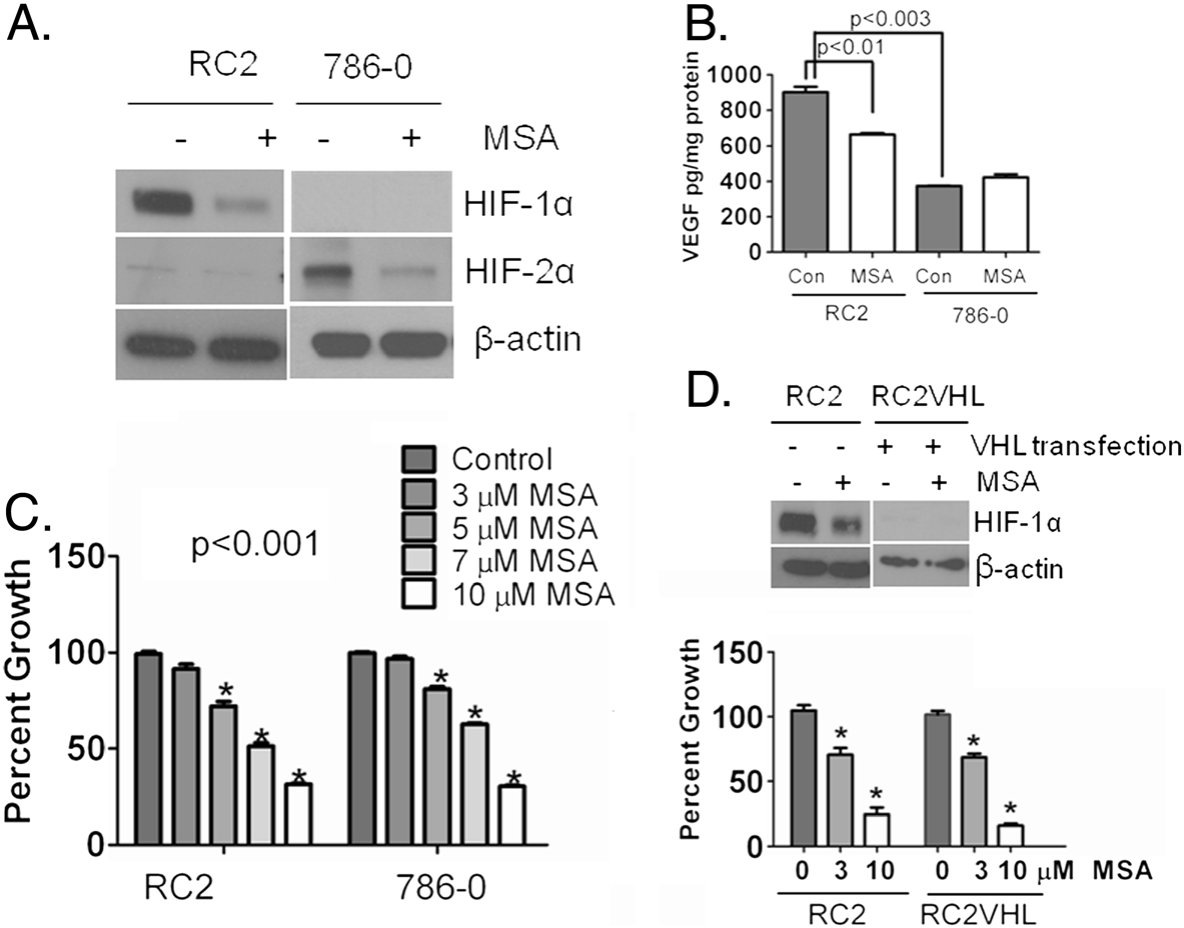
**Effect of selenium treatment on HIF-1α, HIF-2α, VEGF and cell growth.** (**A**) MSA inhibits both HIF-1α and HIF-2α. RC2 cells expressing HIF-1α and 786–0 cells expressing HIF-2α were treated with and without 10 μM MSA for 24 h; HIF-1α and HIF-2α were detected by western blot. β-actin expression was used as a loading control. (**B**) MSA down-regulates secreted VEGF in RC2 cells but not in 786–0 cells. Secreted VEGF was measured by ELISA in ccRCC cells. Cells were treated with and without MSA for 24 h and media were used to measure VEGF in RC2 and 786–0 cells and normalized with protein and expressed as pg/mg protein. Experiment was repeated twice with duplicates and P value < 0.05 was considered as significant. (**C**). HIF-α inhibition by MSA was associated with growth inhibition. Cells were treated with various concentrations MSA (3, 5, 7 and 10 μM) for 24 h. Medium was removed, rinsed and fresh medium was added and allowed to proliferate for 96 h. Cells were fixed and determined the cell survival by SRB assay. Growth inhibition was presented as percent growth inhibition compared to untreated controls. Experiment was repeated twice with 4–5 replicate samples. *p <0.001. (**D**). VHL transfected RC2 cells which do not express HIF-1α were equally sensitive to MSA like RC2 cells which express HIF-1α . Expression of HIF-1α in RC2 and VHL transfected RC2 VHL cells with and without MSA (upper panel). Cytotoxic effects of MSA in RC2 and RC2 VHL cells (lower panel). Cell survival was determined by SRB assay. *p <0.001.

### Inhibition of HIF-1α and HIF-2α by MSA does not translate into comparable downregulation of secreted VEGF, but inhibit the growth of cells

The data presented in Figure 3 demonstrated that treatment with a pharmacological dose of MSA (10 μM) the active metabolite of MSC, resulted in the inhibition of constitutively expressed HIF-1α and HIF-2α in RC2 and 786–0 cells, respectively (Figure 3A). The observed effective inhibition of HIF-α was associated with significant downregulation of secreted VEGF in RC2 cells expressing HIF-1α but not in 786–0 cells expressing HIF-2α (Figure 3B). The data in Figure 3B also indicate that HIF-2α expressing 786–0 cells secreted significantly less VEGF than HIF-1α expressing RC2 cells which might explain the lack of down-regulation of secreted VEGF by MSA. However, under hypoxic conditions, when the secreted VEGF was higher than normoxic conditions, MSA decreased the secreted VEGF levels (data not shown). Irrespective of VEGF levels, inhibition of HIF-α by MSA was associated with significant growth inhibition of RC2 and 786–0 cells (Figure 3C). The results in RC2 cells expressing HIF-1α are consistent with our previous findings of HIF-1α inhibition by MSA resulted in the downregulation of VEGF and growth inhibition in head & neck tumors [22]. The data in Figure 3D shows the VHL restoration degraded HIF-1α in RC2VHL cells but did not alter the sensitivity for MSA under aerobic culture conditions.

### MSA inhibits HIF-1α through post-translational degradation

**Figure 4:**
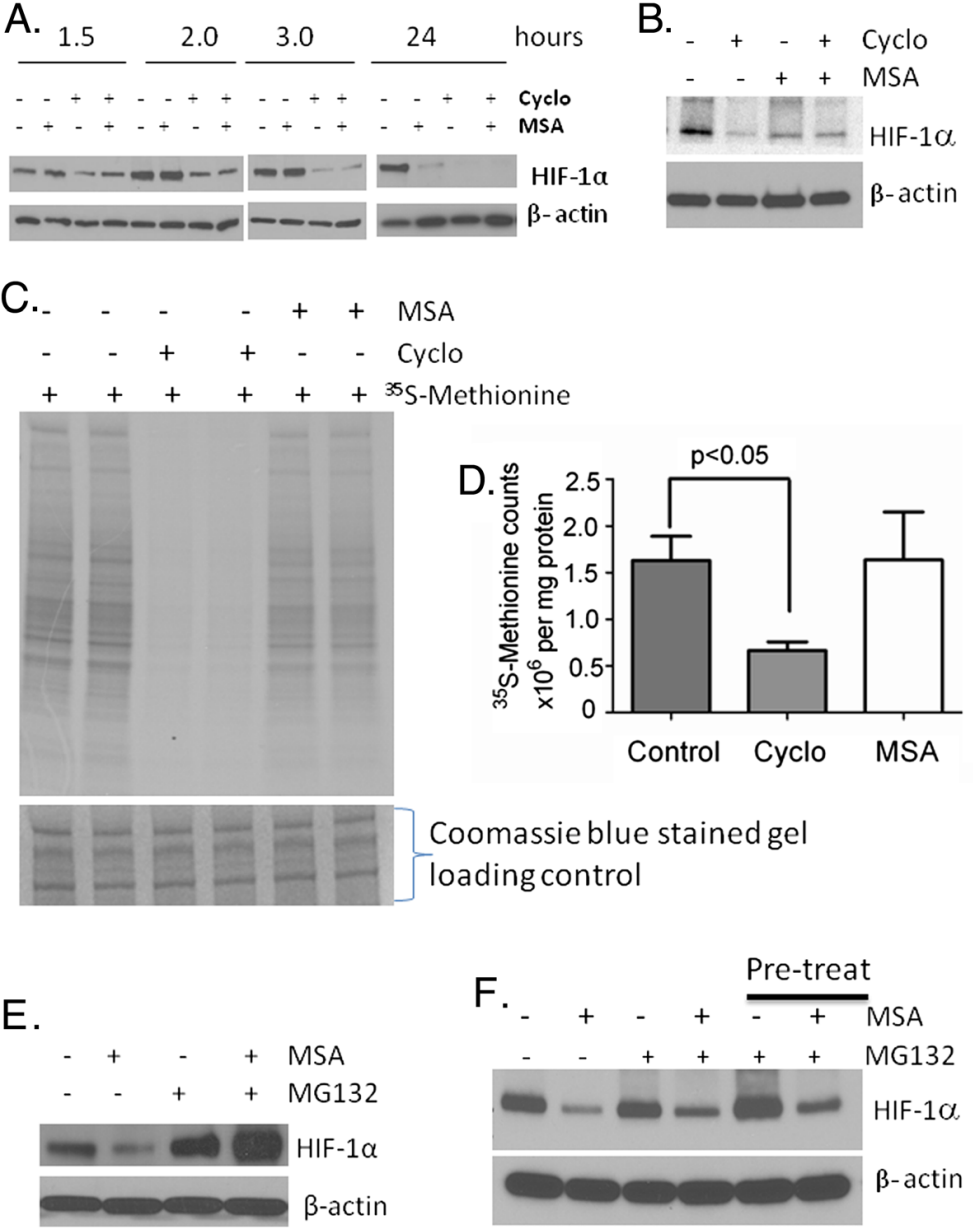
**Effect of MSA on HIF-1α protein synthesis and degradation.** (**A**) HIF-1α synthesis was inhibited by cycloheximide but not by MSA in FaDu cells. FaDu cells were treated with 1 μM MSA and 10 μM cycloheximide alone and in combination for 1.5, 2, 3 and 24 hours at 0.5% oxygen and protein extracts were used to determine HIF-1α by western blot. β-actin expression was used as a loading control. (**B**) Effect of MSA on HIF-1α protein synthesis in RC2 cells. Cells were treated with cycloheximide or MSA alone and in combination for 8 h. HIF-1α was detected by western blot. β-actin was used as loading control (**C**) Incorporation of ^35^ S-Methionine was not affected by MSA in RC2. Cells were treated with cycloheximide or MSA separately in duplicate samples for 5 h and ^35^ S-Methionine (2.3 μCi/ml) was added at the last 1 h of treatment. Protein extracts (20 μg) were used to separate by SDS polyacrylamide gel electrophoresis and detected the incorporated ^35^ S-Methionin by autoradiography. Lower panel showing the coomassie blue stained proteins as loading control. (**D**) Determination of ^35^ S-Methionine radioactivity counts in cycloheximide or MSA treated RC2 cells. Protein extracts (20 μl) were used to detect ^35^ S-methionine radioactivity in the cells by counting in Liquid Scientilator Counter. Total counts were calculated in one milligram of protein and presented the number of counts in millions as compared to untreated control cells. P < 0.05 was considered as significant. (**E**) HIF-1α degradation by MSA is proteasome dependent. FaDu cells which do not express constitutive HIF-1α under normoxic culture conditions were subjected to 0.5% oxygen and treated with 1 μM MSA alone and in combination with 10 μM proteasome inhibitor MG132 for 24 h. Cell extracts were prepared and the expression of HIF-l α was analyzed. Expression of β-actin was used as a loading control. (**F**) Proteasome independent degradation of HIF-1α in VHL mutated RC2 cells. RC2 cells were treated with MSA and MG132 alone and in combination for 8 h. In a separate experiment, 1 h pre-treatment of MG132 followed by 7 h MSA treatment was performed to see the effect of MSA on HIF-1α. Cells were processed to extract protein and HIF-1α levels were determined. Expression of β-actin was used as a loading control.

Three approaches were used to determine whether inhibition of HIF-1α by MSA is at transcriptional or post-translational modification: I) Time dependent inhibition of HIF-1α protein synthesis by MSA was compared to a known protein synthesis inhibitor, cycloheximide (CHX); II) Determine MSA effect on incorporation of ^35^ S-Methionine in protein synthesis, III) Evaluate the effect of a proteasome inhibitor, MG132 alone and in combination with MSA on HIF-1α degradation. The results presented in Figure 4A show that HIF-1α protein synthesis was inhibited by CHX but not by MSA alone in FaDu cells indicating that HIF-1α protein synthesis was not affected by MSA. In RC2 cells CHX inhibited protein synthesis at 4 h (data not shown) and 8 h (Figure 4B). There was some inhibition of HIF-1α with MSA alone at 8 h treatment point which may be due to degradation (Figure 4B). To evaluate precisely whether MSA is inhibiting protein synthesis we have investigated the radiolabeled amino acid incorporation studies with ^35^ S-Methionine, and compared with known protein synthesis inhibitor CHX. The results presented in Figure 4C and D clearly shows that MSA did not inhibit the protein synthesis at 5 h time point in RC2 cells. These results suggest that MSA may inhibit HIF-1α through degradation pathway. To determine whether the selenium mediated degradation of HIF-1α was proteasome dependent, FaDu and RC2 cells were treated with proteasome inhibitor MG132 alone and in combination with MSA and results are shown in Figure 4E and F. The results indicate that while MSA treatment resulted in significant inhibition of HIF-1α, the inhibition of proteasome by MG132 resulted in accumulation of HIF-1α, and this accumulated HIF-1α was not removed by MSA in FaDu cells. In contrast, MSA treatment resulted in degradation of HIF-1α independent of proteasome inhibitor MG132 in RC2 cells. These data suggest that degradation of HIF-1α by MSA was proteasome dependent in FaDu cells but not in RC2 cells.

### Degradation of HIF-1α by MSA is PHD2 dependent and VHL independent

**Figure 5:**
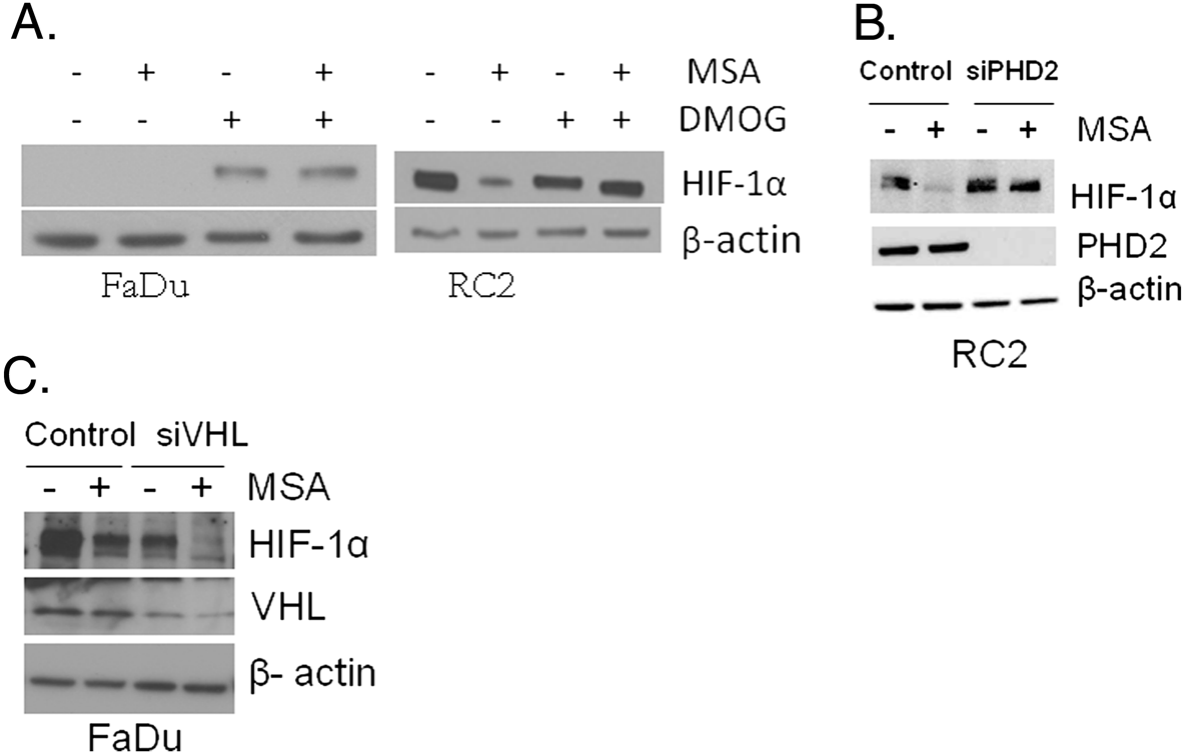
**Role of PHDs in HIF-1α degradation by MSA.** (**A**) Inhibition of PHDs activity by DMOG reversed the degradation of HIF-1α by MSA in VHL active FaDu, and VHL inactive RC2 cells. Cells were treated with 10 μM MSA and 0.5 mM DMOG alone and in combination and HIF-1α was analyzed by western blot. β-actin expression was used as a loading control. (**B**) Gene specific silencing of PHD2 in RC2 cells by siRNA prevented the degradation of HIF-1α by MSA. PHD2 siRNA was transfected with lipofectamine 2000 for 24 h. Cells were treated with and without 10 μM MSA for 24 h and HIF-1α was detected by western blot. β-actin expression was used as a loading control. (**C**) Degradation of HIF-1α by MSA is VHL independent. VHL was inhibited by siRNA in FaDu cells expressing active VHL and treated with MSA to determine the HIF-1α degradation. This experiment was done under 0.5% oxygen level to stabilize HIF-1α in FaDu cells. β-actin expression was used as a loading control.

VHL is inactivated in several human ccRCC [1] and PHD3 is undetectable in all of the 88 ccRCC specimens tested (Figure 1C) and ccRCC cell lines (Figure 2D). To test the hypothesis that the degradation of HIF-1α by MSA is PHD2 dependent, and VHL independent, two approaches were evaluated: i) treat with PHD2 activity inhibitor, DMOG alone and in combination with MSA (Figure 5A) and ii) treat with siRNA against PHD2 (Figure 5B) and VHL (Figure 5C) with the combination of MSA. Since RC2 and 786–0 cells express mutated VHL, we have used FaDu cells which express wild type VHL. HIF-1α is not detectable in FaDu cells under normoxic culture conditions expressing PHD2 and PHD3 [22]. However, inhibition of PHDs activity by DMOG resulted in stable expression of HIF-1α. Treatment of MSA in combination with DMOG did not result in degradation of HIF-1α in FaDu cells expressing PHD2/3 (Figure 5A). In support of these findings, MSA treatment leads to degradation of HIF-1α in RC2 cells expressing PHD2 protein with nonfunctional VHL and this degradation is reversed in combination with DMOG (Figure 5A). Consistent with these findings, inhibition of PHD2 by siRNA did not resulted in the degradation of HIF-1α by MSA in RC2 tumor cells expressing constitutive HIF-1α with mutated VHL (Figure 5B). The data in Figure 5C demonstrated that inhibition of VHL by siRNA did not prevent HIF-1α degradation by MSA in FaDu cells expressing functional VHL. Collectively, the data is consistent with the hypothesis that degradation of HIF-1α by a pharmacological dose of MSA is PHD2 dependent, and VHL independent. 

### Degradation of HIF-2α by MSC is associated with antitumor activity in 786–0 tumor xenografts

**Figure 6:**
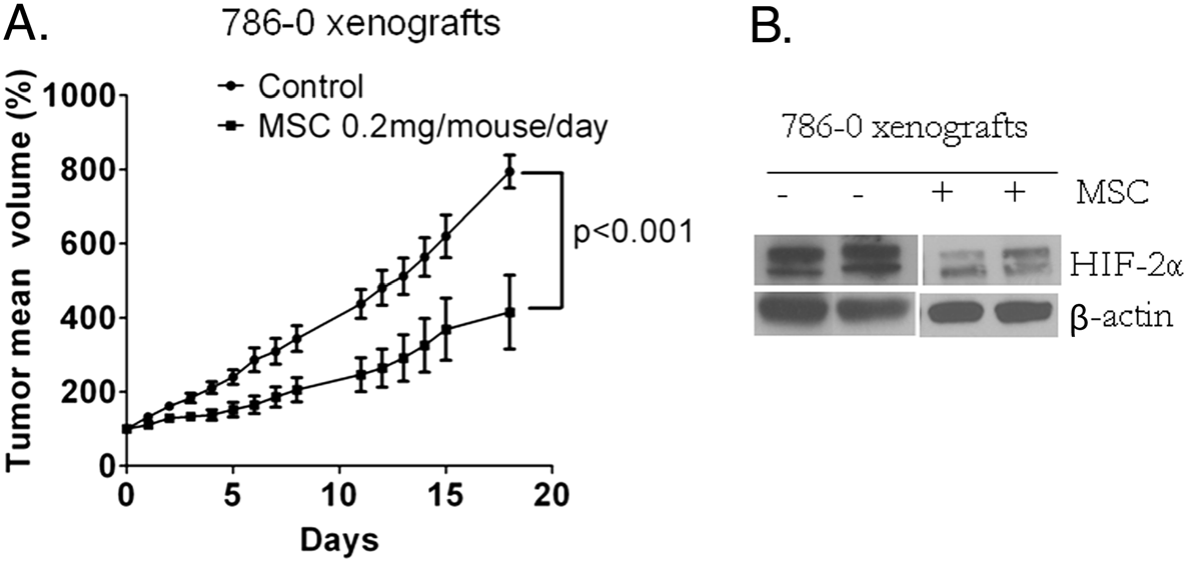
**MSC effect on tumor growth and HIF-2α expression in 786–0 ccRCC xenografts.** (**A**) Inhibition of ccRCC tumor growth by MSC in xenografts. HIF-2α expressing 786–0 cells (10 million) were transplanted into nude mice for establishing the xenografts. Small pieces (~50 mg) of tumor tissues were transplanted subcutaneously into nude mice and treatment began when tumor weighed 200–250 mg. Mice were randomized and divided into two groups, each containing 5 mice. One group was treated with saline and the other group was treated with MSC (0.2 mg/mouse/day; the optimal nontoxic dose) daily for 18 days. Tumor volume was measured daily. (**B**) HIF-2α is inhibited by the therapeutic dose of MSC in 786–0 xenografts. Tumor xenografts collected after 18 days of the MSC treatments and processed to extract protein and HIF-2α levels were assessed by western blot. β-actin expression was used as a loading control.

To confirm that inhibition of HIF-2α by a nontoxic dose of MSC will translate into therapeutic benefits, 786–0 xenografts expressing constitutively active HIF-2α were treated orally daily with 0.2 mg/mouse/day MSC for 18 days. The data presented in Figure 6 showed that MSC treatment resulted in significant inhibition of tumor growth (Figure 6A) which was associated with inhibition of HIF-2α (Figure 6B). These data are consistent with the previous finding from this laboratory demonstrating that the inhibition of HIF-1α by MSC resulted in significant antitumor activity against FaDu tumor xenografts [22]. 

## Discussion

The expression of PHD2/3, the main regulators of HIF-α has not been investigated in primary human ccRCC using double immunohistochemical staining to detect these proteins simultaneously in consecutive sections of the same tumors. In this study, we have demonstrated low incidence, distribution and staining intensity of PHD2, deficient PHD3 protein, and high HIF-α incidence, distribution and intensity in 88 primary ccRCC cancers compared to head & neck and colorectal cancers (Figure 1A, B and C). Furthermore, like clinical samples, the two ccRCC cell lines (RC2 and 786–0) used for mechanistic studies were deficient in PHD3 protein (Figure 2D) but not mRNA (Figure 2B). The high incidence of HIF-α in ccRCC has been partially linked to the mutation of VHL gene. The VHL gene mutation incidence varies from 19.6 to 89.4% in ccRCC [32,33] and the majority of reports show 30-60% mutation incidence [34]. Furthermore, the up-regulation of both HIF-1α (88.2%) and HIF-2α (100%) with only 39.1% VHL mutations was found in ccRCC showing the VHL independent up-regulation of HIF-α in many cases [34]. Our results suggest a role for PHD2/3 in addition to the well documented VHL mutations in the constitutive expression of HIF-α in ccRCC. A recent report showed the silencing of PHD3 expression by CpG methylation in the promoter region of human cancer cell lines including renal cancer, prostate, breast and melanoma [12], and in plasma cells and B-cell lymphoma, suggesting PHD3 as a potential biomarker [35]. In addition, Astuli et al., [36] found the absence of pathogenic mutations in PHD1, 2 and 3 that could cause renal cell carcinoma. Our western blot analysis showed very weak expression of PHD3 protein compared to PHD2 (Figure 2C) in two representative primary tumor cases. This weak signal may be derived from the normal stromal cells expressing PHD3 [9]. These results suggest that there may be some epigenetic regulation of PHD3 expression in ccRCC that might lead to the degradation or inhibition of PHD3 protein. A recent clinical study showed a positive correlation between decreased PHD3 expression and aggressive type of breast tumors [37]. Similarly, the lack of expression or low incidence/intensity of PHD3 may contribute to the aggressiveness of ccRCC tumors. Thus, the agents that enhance HIF-α degradation by PHD2, independent of PHD3 expression may offer treatment modality that could affect resistance and clinical outcome.

This laboratory is the first to show that therapeutic dose of selenium as highly effective inhibitor of both constitutively expressed HIF-1α, HIF-2α in ccRCC (Figure 3A and 6B) and hypoxia induced HIF-1α in head & neck cancer [22]. Consistent with our data, published results show the degradation of constitutively expressed HIF-1α in prostate cancer [38] and hypoxia induced HIF-1α in B-cell lymphoma [39] by selenium. These findings show that both hypoxia induced and constitutively expressed HIF-α are inhibited by selenium suggesting that selenium could inhibit growth of tumors expressing HIF-1α, HIF-2α or both. HIF-α transcriptionally regulated gene, VEGF, is regulated by MSA in renal cancer cells (Figure 3B). MSA treatment leads to the down-regulation of secreted VEGF in HIF-1α expressing RC2. The lack of MSA effects on secreted VEGF in 786–0 cells could be due to low levels of secreted VEGF in these cells. To our surprise we did not see difference in cytotoxic effects of MSA in RC2 and RC2VHL cells even though there is a marked difference in HIF-1α levels in these cells under normoxic culture conditions. This may be due to the other effects of MSA in these particular cells with VHL transfection. VHL being a multifunctional adaptor molecule involved in the inhibition of HIF-α independent and dependent cellular processes [40]. The cytotoxic effects of MSA in RC2VHL cells may be through VHL interacting proteins. Our data demonstrate that selenium main target HIF-α is degraded by PHD dependent and VHL independent, but some of our unexpected findings with VHL transfected RC2 cells indicate that VHL transfection may influence the cytotoxic effects of MSA independent of HIF-1α by currently unclear molecular mechanism.

We have demonstrated HIF-α inhibition by selenium as a post-translational degradation mechanism. As shown in the Figure 4A and B, MSA did not affect HIF-α protein synthesis. In a separate experiment, we have demonstrated that the overall protein synthesis was not altered by MSA using the ^35^ S-Methionine incorporation studies (Figure 4C and D). The proteasome inhibitor MG132 reversed the degradation of HIF-α by MSA in FaDu cells (Figure 4E) demonstrating the proteasome dependent degradation. In contrast, in RC2 cells proteasome inhibition did not reverse the degradation of HIF-1α by MSA suggest that in VHL mutant cells MSA may be degrading HIF-1α through proteasome independent pathway. Further detailed mechanistic studies need to be performed to investigate how MSA is degrading HIF-α in the absence of VHL in ccRCC. Our results also show that MSA is unable to degrade HIF-1α stabilized by DMOG, an inhibitor of PHDs activity (Figure 5A). DMOG inhibits PHD activity by competing with 2-oxoglutarate, a cofactor for PHDs activity. In addition, gene specific inhibition of PHD2 also prevented the degradation of HIF-1α by MSA (Figure 5B). Furthermore, we have confirmed VHL independent degradation of HIF-1α by silencing of VHL with siRNA in VHL positive FaDu cells (Figure 5C). As reported in the literature, VHL knockdown did not lead an increase of HIF-1α in FaDu cells under hypoxic conditions [41]. These results indicate that selenium utilizes a unique pathway for HIF-1α degradation through PHD2 dependent and VHL independent degradation mechanism. Future studies are warranted to investigate specific function of PHD2 that might be altered by selenium leading to the degradation of HIF-α through another ligase independent of VHL.

Our recent report [22] and study by Sinha et al., [38] demonstrated stabilization of PHDs by MSA leads to the degradation of HIF-1α. HIF-1α degradation through VHL dependent and independent pathways is known. Under aerobic conditions, HIF-1α is hydroxylated at 402 and 564 proline molecules by PHDs and recognized by VHL and further degraded by proteasome [42,43]. HIF-1α is also degraded without PHD through a small ubiquitin-like modifier (SUMO)ylation that allows the binding of VHL to further degrade HIF-1α by proteasome [44]. There has been growing evidence for VHL independent degradation of HIF-1α through histone deacetylases (HDACs) inhibition [28,39], heat shock protein 90 (HSP90) [45,46], the hypoxia associated factor (HAF) [47] and an undescribed cullin-independent proteasome degradation pathway [29,48].

Based on the demonstrated low incidence of PHD2, lack of PHD3 protein and high incidence of HIF-α in ccRCC, we expect that HIF-α mediated drug resistance is particularly important in this type of cancer. Therefore, decreasing HIF-α expression in ccRCC cells seems to be an important new strategy in order to sensitize tumor cells to the currently used standard therapy. We found MSA treatment lead to 786–0 tumor growth inhibition which correlated with reduced HIF-2α protein levels (Figure 6). It is important to indicate that although HIF-1α role in drug resistance has been widely evaluated [49], to date, efforts have been focused on the development of agents that would effectively inhibit HIF-1α synthesis [50-52]. MSC represents a new type of HIF-α inhibitor by enhancing the degradation, but not affecting the synthesis of HIF-α. Currently, it is difficult to predict what approach of HIF-α inhibition combined with chemotherapy will improve the cancer therapy. Furthermore, utilization of clinically more relevant orthotopic imageable mouse models [53-55] would be more appropriate for further development of MSC as HIF-α inhibitor in ccRCC.

## Conclusions

We have demonstrated that low incidence of PHD2 and deficiency of PHD3 protein associated with high incidence of HIF-α in ccRCC. Both HIF-1α and HIF-2α are inhibited by MSC through PHD2 dependent and VHL independent degradation mechanism. Furthermore, HIF-2α degradation by MSC leads to inhibition of the growth of ccRCC tumor xenografts without toxicity. Thus, our data supports further evaluation of MSC as a HIF-α inhibitor in combination with multikinase inhibitors, like sunitinib, to determine their efficacy in ccRCC xenograft model.

## Abbreviations

CCRCC: Clear cell renal cell carcinoma; HIF-1α: Hypoxia inducible factor-1 alpha; HIF-2α: Hypoxia inducible factor 2 alpha; HIF-α: HIF-1α and HIF-2α; PHD: Prolyl hydroxylase; VHL: Von-Hippel-Lindau; VEGF: Vascular endothelial growth factor; SiRNA: Small interfering RNA; MSC: Se-Methylselenocysteine; MSA: Methylselenic acid; DMOG: Dimethyloxallyl glycine; MG132: N-[(phenylmethoxy)carbonyl]-L-leucyl-N-[(1 S)-1-formyl-3-methylbutyl]-L-leucinamide; TMA: Tissue microarray; DBBR: Data bank and bioRepository; EGLN1: Egg-laying-defective nine1.

## Competing interests

The authors declare that they have no competing interests.

## Authors’ contributions

SC: Conception, design and collection of data, manuscript writing, data analysis and interpretation. TB: Design, collection, analysis and interpretation of *in vitro* data. KT: Analysis and interpretation of TMAs immunohistochemical data. SC and FAD: Collection, analysis and interpretation of animal experiments data. RP: Provision of patients samples, analysis and interpretation of data. YMR: Conception, design, and manuscript writing. All authors read and approved the final manuscript.

## Pre-publication history

The pre-publication history for this paper can be accessed here:

http://www.biomedcentral.com/1471-2407/12/293/prepub
